# Automatic lung segmentation in chest X-ray images using improved U-Net

**DOI:** 10.1038/s41598-022-12743-y

**Published:** 2022-05-23

**Authors:** Wufeng Liu, Jiaxin Luo, Yan Yang, Wenlian Wang, Junkui Deng, Liang Yu

**Affiliations:** 1 Henan University of Technology, Zhengzhou, 450001 China; 2 Nanyang Central Hospital, Nanyang, 473009 China

**Keywords:** Computer science, Computational biology and bioinformatics

## Abstract

The automatic segmentation of the lung region for chest X-ray (CXR) can help doctors diagnose many lung diseases. However, extreme lung shape changes and fuzzy lung regions caused by serious lung diseases may incorrectly make the automatic lung segmentation model. We improved the U-Net network by using the pre-training Efficientnet-b4 as the encoder and the Residual block and the LeakyReLU activation function in the decoder. The network can extract Lung field features efficiently and avoid the gradient instability caused by the multiplication effect in gradient backpropagation. Compared with the traditional U-Net model, our method improves about 2.5% dice coefficient and 6% Jaccard Index for the two benchmark lung segmentation datasets. Our model improves about 5% dice coefficient and 9% Jaccard Index for the private lung segmentation datasets compared with the traditional U-Net model. Comparative experiments show that our method can improve the accuracy of lung segmentation of CXR images and it has a lower standard deviation and good robustness.

## Introduction

Among the existing medical imaging methods, X-ray is one of the most commonly used diagnostic technology as it is widely available, low cost, non-invasive, and easy to acquire^1,2^. Chest radiography is the most popular and important imaging modality used to diagnose various pulmonary diseases. Applying deep learning in medical imaging can help medical experts carry out screening and diagnosis and reduce their burden^3,4^. Segmentation of the lung becomes challenging due to several reasons: (1) non-pathological changes: the shape and size of the lung vary with age, gender, and heart size; (2) pathological changes: the opacity caused by severe lung disease reaches a high-intensity value^5^; (3) foreign body coverage, such as the lung field, is obscured by the patient's clothes or medical equipment (pacemaker, infusion line, medical catheter)^6^. Most of the reported work on lung segmentation is based on mild lesions or healthy CXR images. Therefore, it is necessary to verify the ability of the lung segmentation model on complex CXR images. So we randomly screened 2785 CXRs from the NIH (National Institute of Health) Chest X-ray dataset^7^ (https://www.kaggle.com/nih-chest-xrays/data) and invited experienced radiologists to label the lung field manually. In particular, these 2785 images contain some severe lung diseases. In addition, we also designed an excellent lung field semantic segmentation model, which is structured by U-Net^8^ and uses the Efficientnet-b4 pre-training model as the backbone (https://github.com/2112942597/2985).

In related literature, many methods have been proposed for automatic lung segmentation. These methods have a wide application prospect. It can be divided into two categories: traditional methods based on machine learning and methods based on deep learning. Traditional lung segmentation methods do not rely on the dataset labeled by professional radiologists, so they are easy to implement. But the lung boundaries obtained may not be optimum due to the heterogeneity of lung field shapes. The accuracy of this kind of algorithm is far lower than that of neural network modeling^6,9^.

In recent years, with the progress of computer image processing ability and the continuous enrichment of datasets, deep learning technology has achieved good results in medical image analysis^10–12^. In semantic segmentation technology, the chest radiograph is used as the input of a deep neural network, which classifies each pixel into lung region or non-lung region^13^.

Hwang et al.^14^ proposed a model based on the atrous convolution architecture for accurate lung segmentation. Their algorithm was tested on JSRT^15^ and Montgomery County (MC) datasets^16^, and the dice coefficients were 0.9800 and 0.9640, respectively. Rahul et al.^17^ used full convolution neural networks to segment the lung field of JSRT and MC datasets, with an average accuracy of 98.92% and 97.84%, respectively. Mittal et al.^18^ modified the upsampling part of the famous SegNet architecture^19^ and trained it on the JSRT dataset. When tested on JSRT and MC datasets, their model achieves 98.73% accuracy.

Ngo et al.^20^ propose a new methodology for lung segmentation in CXR using a hybrid method based on a distance regularized level set and deep structured inference. Their average accuracy on JSTR varies from 94.8 to 98.5%. Rashid, Ret al.^21^. proposed the full convolution network for lung segmentation. The average accuracy on JSRT, MC, and local data sets are 97.1%, 97.7%, and 94.2%, respectively. Ching Sheng change et al. annotated the lung region of the NIH chest X-ray data set, and then performed semantic segmentation^22^. They achieved a 94.9% Jaccard index score. Souza, J. et al.designed an automatic lung segmentation and reconstruction method based on a depth neural network^23^. Based on the deep neural network, Lamin Saidy et al.introduce the knowledge of graphic morphology to solve the problem of fragments in lung segmentation^24^.

## Methods

In image segmentation tasks, especially medical image segmentation, U-Net^8^ is undoubtedly one of the most successful methods. Compared with FCN^25^, SegNet^19^, and Deeplab^26^, U-Net uses skip connection in the same stage instead of direct supervision and loss back transmission on high-level semantic features. It ensures that the finally recovered feature map integrates more low-level features and enables the fusion of elements of different scales. Thus, multi-scale prediction and deep supervision can be carried out. Upsampling also makes the information, such as the restored edge of the segmented image, finer. A challenge of deep learning for medical image processing is that it often provides few samples, and U-Net still performs well under this limitation. Based on these advantages, we choose U-Net as the framework of the automatic lung segmentation model. The input size of the model is 256 * 256 * 3, and the output size is 256 * 256 * 1—our experiment with Imagenet's pre-trained base networks. The network architecture used in this work has five coding layers and five decoding layers. The encoder is Efficientnet-b4 pre-trained on the Imagenet.

The innovation of our model mainly lies in the decoding block. The decoder consists of five blocks; Each decoding layer includes a dropout layer, a two-dimensional convolution and padding layer, and finally, two residual blocks and a LeakyReLU. We also try to concatenate three residual blocks in each decoding block, but the model's performance is not improved. The function of the dropout layer is to improve the generalization ability of the model and prevent the model from overfitting. The two-dimensional convolution layer continues to extract image information. Two residual blocks^27^ can prevent the “vanishing gradient” and make information spread better.

**Figure 1:**
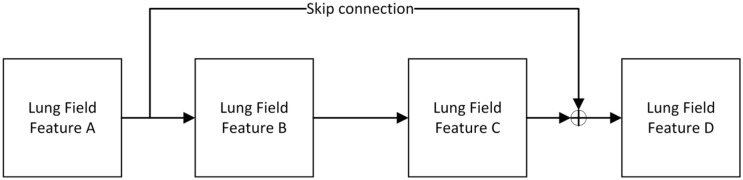
The principle of the residual block.

Residual block is the most important module in Resnet^28^. It adds a quick connection between the input and output of network layers. In other words, it directly adds the original information and output without any change. The deeper the network is, the more obvious the "vanishing gradient," and the training effect of the network will not be very good. But now, the shallow network can not significantly improve the network performance. That's a contradictory problem, but the residual block effectively solves the contradiction of avoiding the "vanishing gradient" when deepening the network. Figure 1 and Formulas (1–3) show how this is achieved. Even if the gradient attenuation occurs in the backward propagation of A-B-C, the gradient at D can still be directly transmitted to A; that is, the cross-layer propagation of the gradient is realized. From the perspective of gradient size, no matter how deep the network structure is, the residual network can maintain a large value of the weight close to the data layer (input) to alleviate the vanishing gradient.

LeakyReLU^29^ was used as the activation function. The function of LeakyReLU is very similar to that of ReLU. The only difference is in the part where the input is less than 0. The value of the part where the input of ReLU is less than 0 is 0, while the value of the part where the input of LeakyReLU is less than 0 is negative and has a slight gradient. Suppose RelU is used as the activation function of the middle layer when the gradient of the backpropagation process is 0. In that case, the corresponding weight and bias parameters cannot be updated this time. Then the neuron can no longer learn. This phenomenon is called "neuron death." So we use LeakyReLU as the middle layer's activation function to avoid this problem. Finally, we apply a 1 × 1 convolution layer and then use the "Sigmoid" activation function to output the mask.

1 $$\frac{\partial L}{{\partial X_{{A_{out} }} }} = \frac{\partial L}{{\partial X_{Din} }}\frac{{\partial X_{Din} }}{{\partial X_{{A_{out} }} }}$$

2 $$X_{Din} = X_{{A_{out} }} + C\left( {B\left( {X_{{A_{out} }} } \right)} \right)$$

3 $$\frac{\partial L}{{\partial X_{{A_{out} }} }} = \frac{\partial L}{{\partial X_{Din} }}\left[ {1 + \frac{{\partial X_{Din} }}{{\partial X_{C} }}\frac{{\partial X_{C} }}{{\partial X_{B} }}\frac{{\partial X_{B} }}{{\partial X_{{A_{out} }} }}} \right]$$

### Loss function

Utilizing the dice loss as the loss function.

4 $$DSC_{{\left( {A,B} \right)}} = \frac{{2\left| {A \cap B} \right|}}{\left| A \right| + \left| B \right|} = \frac{TP}{{2TP + FP + FN}}$$

5 $$L_{dice\_loss} = 1 - dsc$$

### Training details and hyper-parameters

**Figure 2:**
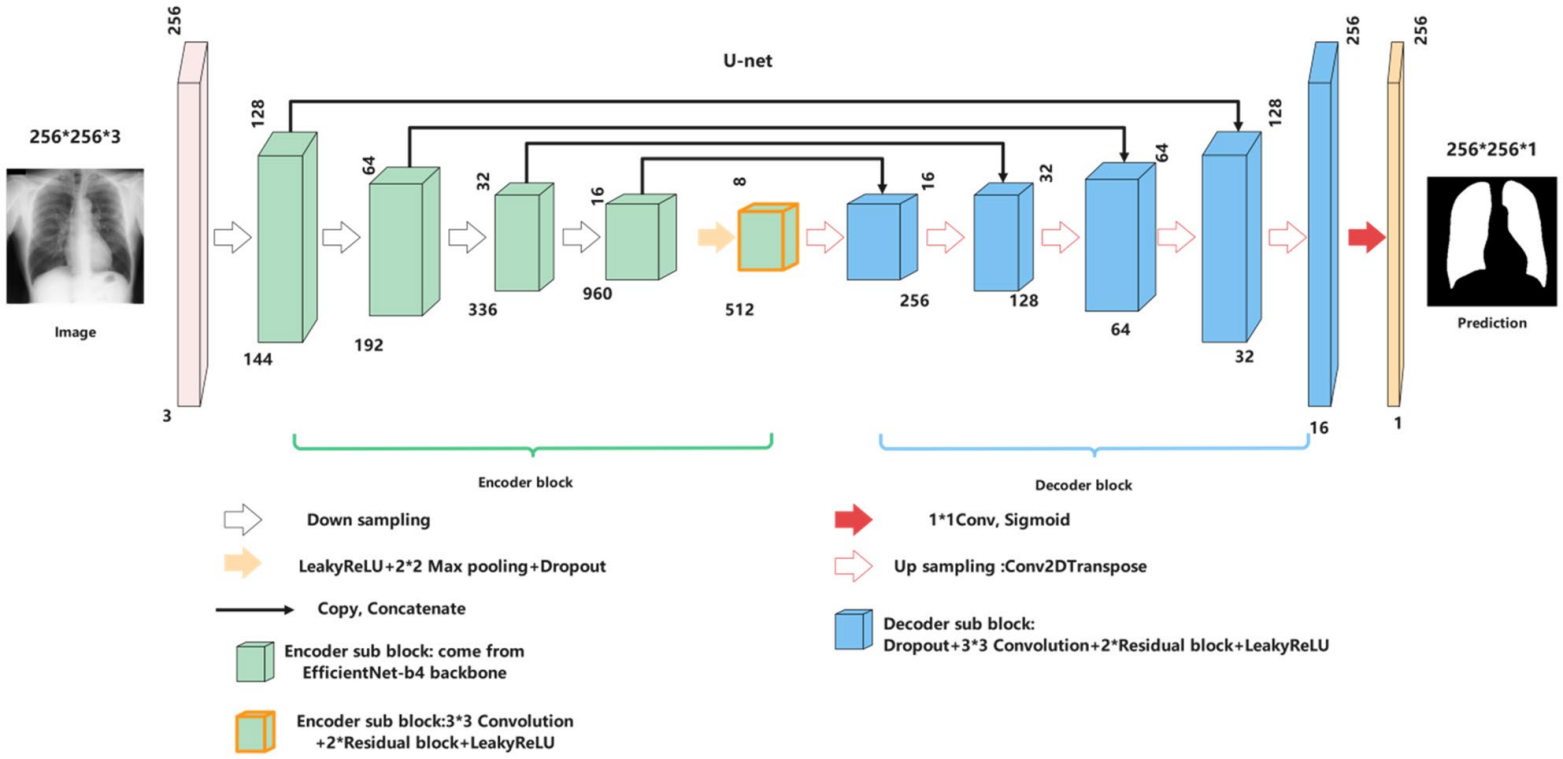
The architecture of U-Net with EfficientNet-b4 Encoder.

**Figure 3:**
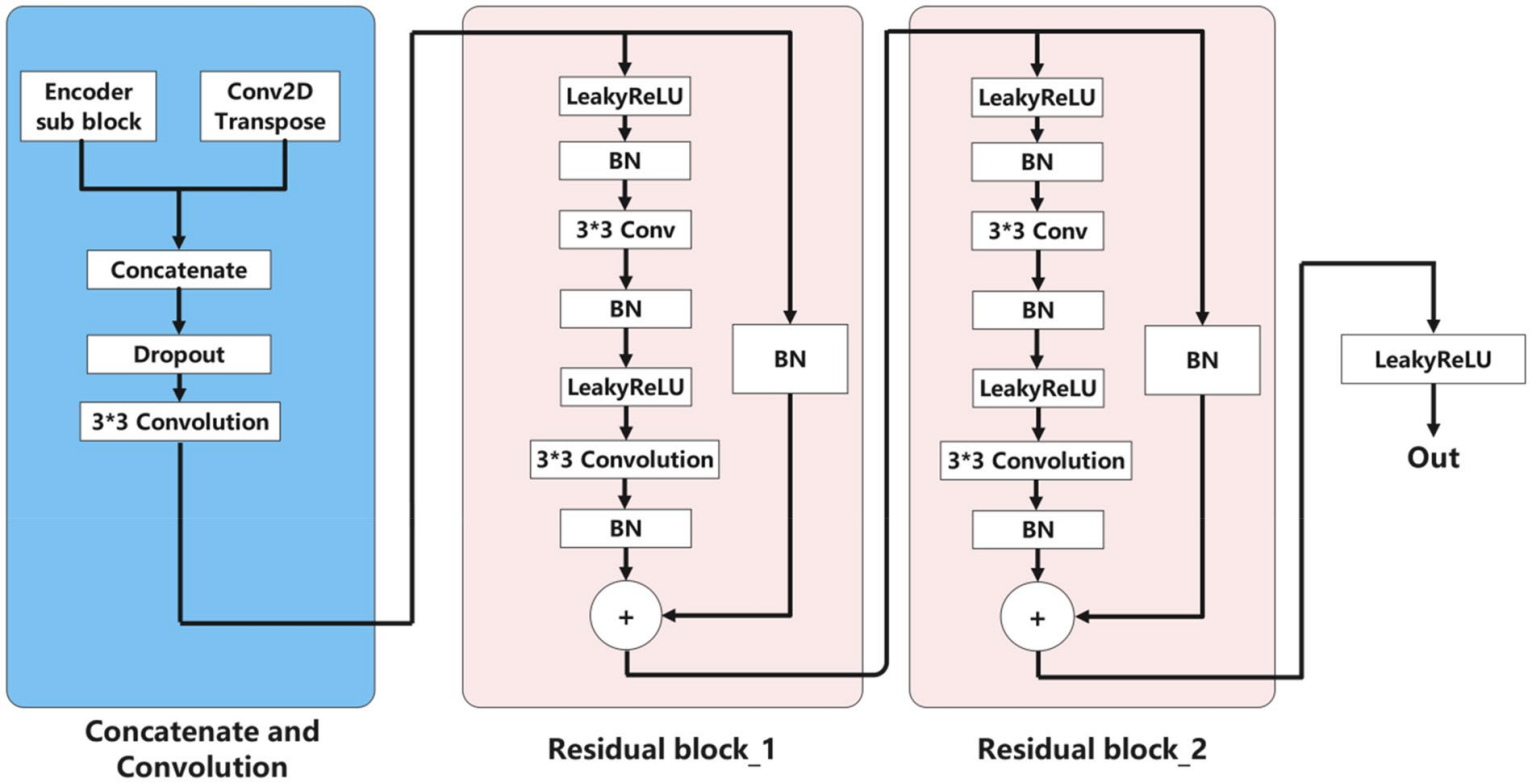
Encoder sub-block as shown.BN refers to Batch Normalization.

The initial learning rate of the model is set to 0.0002. The batch size is set to 64. Max epochs are set to 70. The model is not improved every ten epochs, and the learning rate is automatically reduced by half. Figures 2 and 3 show the architecture of our model and the detail of the decoder sub-block. We used the data enhancement tool "Albumentations" (https://github.com/albumentations-team/albumentations). It is a fast training data enhancement library for OpenCV. It has a very simple and powerful interface that can be used for various tasks (segmentation and detection). It is easy to customize and convenient to add other frameworks. It can convert the data set pixel by pixel, such as blur, downsampling, Gaussian point making, Gaussian blur, dynamic blur, RGB conversion, random atomization, etc.; In this work. We use random gamma, blur, horizontal flip, normalization, and other data enhancement methods. The specific model code and data enhancement code have been open-source on GitHub. The network was trained using two-thirds of the images, in which 20% of the data were reserved for validating the training process and tuning the models, and the image size was adjusted to 256 * 256. Our model is trained using the Tensorflow-2.40 platform on NVIDIA GeForce RTX 3060 GPU with Intel CPU Core i5-11600 K@ 3.9 GHz, 32 GB RAM.

### Lung segmentation in benchmark datasets (JSRT&MC)

The Japanese Society of Radiological Technology creates the JSRT dataset^15^ in collaboration with the JapaneseRadiological Society. It contains 247 CXRs of 2048 × 2048 resolution. Of the 247 images, 93 are normal, and 154 are abnormal, with TB manifestations. These images are stored in PNG format with 2048 × 2048 pixels having 12 bits grayscale. The abnormality of images is graded from extremely subtle to obvious.

The Montgomery County(MC) dataset^16^ is created by the Department of Health and Human Services, Montgomery County, Maryland, USA. The dataset contains 138 CXR images, including 80 healthy cases and the remaining 58 are cases of tuberculosis. They can also be made available in Dicomformat upon request. The size of the X-rays is either 4020 × 4892 pixels.

**Figure 4:**
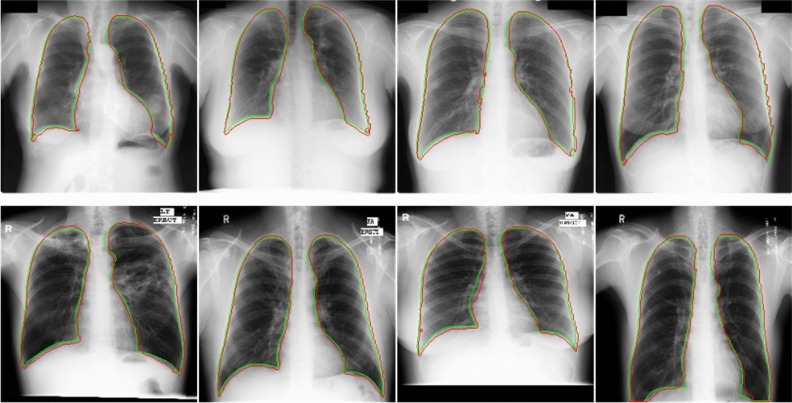
The first row is the lung segmentation result in the JSRT dataset, and the second row is the segmentation results in the MC dataset. The ground-truth lung boundary is depicted in green, and the automatically segmented lung boundary by our method is presented in red color.

Figure 4 shows the performance of our lung segmentation model in two benchmark datasets. Our model generally achieves excellent segmentation scores in dealing with two benchmark datasets (mild disease, no foreign body occlusion, high image quality). That shows the reliability of our dataset and model. However, since these two public datasets do not contain complex chest radiographs, we also need to verify the model's ability to process difficult chest radiographs on Haut datasets. The Jaccard Index is an extremely important metric to evaluate our method because it represents the rate of lung pixels correctly segmented, which is directly related to the objective of our work. Data enhancement techniques are used to generate new images to compensate for the limited size of the dataset. Horizontal Flip and Rotation are transformations used to create new images (Tables 1 and 2).

**Table 1:** Lung segmentation results on the JSRT dataset (mean ± standard deviation).

	Accuracy (%)	Specificity (%)	Sensitivity (%)	Dice coefficient (%)	Jaccard Index (%)
Method 1	97.92 ± 0.92	97.22 ± 1.77	91.60 ± 3.60	95.22 ± 0.85	89.34 ± 1.54
Method 2	98.67 ± 0.44	98.51 ± 1.38	94.83 ± 2.80	95.97 ± 0.65	91.05 ± 1.20
Method 3	98.45 ± 0.52	98.43 ± 1.43	94.35 ± 2.54	96.50 ± 0.55	92.20 ± 1.02
Method 4	98.89 ± 0.36	98.52 ± 0.81	97.28 ± 1.06	97.23 ± 0.16	94.68 ± 0.30
Method 5	**98.55 ± 0.29**	**98.56 ± 0.44**	**98.40 ± 0.87**	**97.92 ± 0.14**	**95.73 ± 0.27**
Method 6	98.55 ± 0.28	98.55 ± 0.44	98.38 ± 0.88	97.90 ± 0.13	95.70 ± 0.26

Significant values are in bold.

**Table 2:** Lung segmentation results on the MC dataset (mean ± standard deviation).

	Accuracy (%)	Specificity (%)	Sensitivity (%)	Dice Coefficient (%)	Jaccard Index (%)
Method 1	97.71 ± 0.95	96.98 ± 1.56	91.53 ± 3.71	95.29 ± 0.72	89.69 ± 1.31
Method 2	97.67 ± 0.44	97.51 ± 1.38	94.53 ± 3.29	95.82 ± 0.60	90.84 ± 1.10
Method 3	98.25 ± 0.41	98.45 ± 1.26	94.35 ± 2.48	96.16 ± 0.43	91.82 ± 0.79
Method 4	98.76 ± 0.47	98.22 ± 0.93	97.28 ± 1.22	97.38 ± 0.19	94.56 ± 0.32
Method 5	**98.94 ± 0.33**	**99.33 ± 0.25**	**97.52 ± 0.95**	**97.82 ± 0.19**	**95.55 ± 0.28**
Method 6	98.96 ± 0.37	99.30 ± 0.27	97.50 ± 0.87	97.83 ± 0.18	95.53 ± 0.27

Significant values are in bold.

Method 1: U-net architecture + Efficientnet-b4 encoder.

Method 2: U-net architecture + Efficientnet-b4 encoder + LeakyReLU.

Method 3: U-net architecture + Efficientnet-b4 encoder + Residual block.

Method 4: U-net architecture + Efficientnet-b4 encoder + LeakyReLU. +Residual block.

Method 5: U-net architecture + Efficientnet-b4 encoder + two Residual blocks + LeakyReLU.

Method 6: U-net architecture + Efficientnet-b4 encoder + three Residual blocks + LeakyReLU.

### Datasets used in the experiment

The NIH Chest X-ray Dataset comprises 112,120 X-ray images with disease labels from 30,805 unique patients. There are 15 classes (14 diseases and "No findings"). Images can be classified as "No findings" or one or more disease classes, showing 14 common thoracic pathologies. NIH Chest X-ray dataset itself does not contain lung field labels. We randomly selected 2785 samples and invited doctors (Wenlian Wang and Junkui Deng from Nanyang Central Hospital) to label the image's lung fields. We call this new dataset Haut. The Haut dataset contains some chest radiographs that are seriously blurred, obscured, and deformed. Haut dataset contains 1647 normal individuals and 1138 patients with CXR’s lung field masks, including 193 with Infiltration, 111 with Atelectasis, 78 with Effusion, 65 with Nodule, 54 with Mass, 43 with Pneumothorax, 37 with Cardiomegaly, 37 with pleural thickening, 34 with Fibrosis, 25 with Consolidation, 21 with Emphysema,11 with Edema, 10 with Pneumonia, 2 with Hernia, and 417 with Multiple diseases (including any two or more diseases above). To use Efficientnet-b4, the images were downsized to 256 × 256 pixels as a pre-processing step. The following Table 3 shows the detail of the datasets used in the experiment.

**Table 3:** Three lung segmentation datasets were used in this experiment.

	JSRT	MC	Haut (private dataset)
Healthy cases	93	80	1647
Unhealthy cases	Lung nodules:154	Tuberculosis:58	Multiplediseases:417 Infiltration:193 Atelectasis:111 Effusion:78 Nodule:65 Mass:54 Pneumothorax:43 Cardiomegaly:37 PleuralThickening:37 Fibrosis:34 Consolidation :25 Emphysema:21 Edema:11 Pneumonia:10 Hernia:2
Total	247	138	2785

### Computer graphics morphological repair

**Figure 5:**
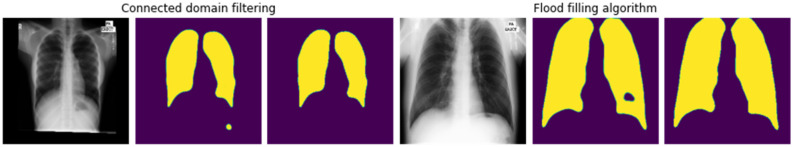
The connected domain filtering algorithm and flood filling algorithm.

Considering that fragments (False Positive, FP) and holes (False Negative, FN) will appear in the lung segmentation of some CXR images, we used two optimization methods to eliminate false positives and false negatives in segmentation. For fragment (FP), we use the connected domain filtering algorithm. Only the two largest connected regions in the image (corresponding to the left and right lungs of the human body) are retained, and small fragments are filtered out. For holes (FN), we use the flood filling algorithm to repair them. The following Fig. 5 shows the specific functions of these two algorithms.

### Lung segmentation in complex case (Haut)

**Figure 6:**
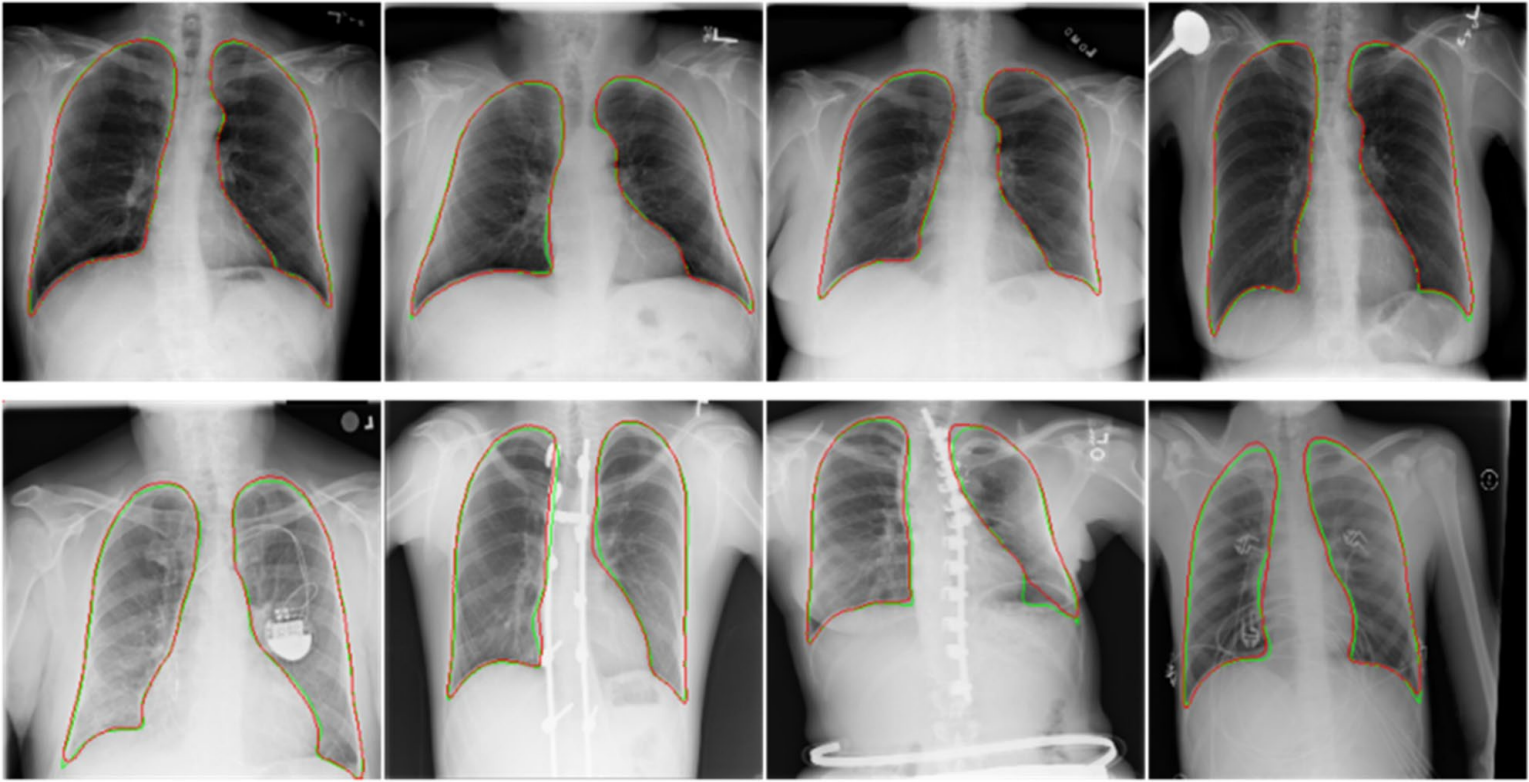
As shown in the figure above, we comprehensively evaluated the Haut dataset. Green represents the real lung field and red represents the lung field predicted by the model. The first line belongs to healthy or mild symptoms, and the effect of lung segmentation is very good. The second line is that foreign bodies (various medical devices) block the lung field, and the segmentation effect is relatively poor.

**Figure 7:**
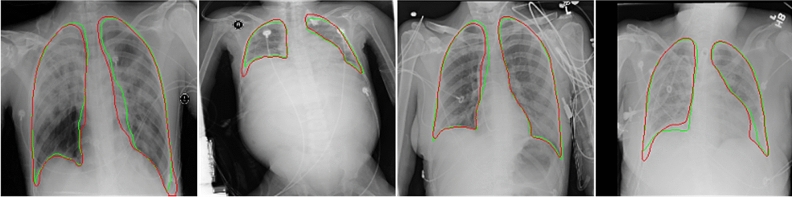
The above is the result of lung segmentation in severe disease (blurred lung area caused by disease) and distorted lung. In those cases, the lung segmentation score is the lowest. The ground-truth lung boundary is depicted in green, and the automatically segmented lung boundary by our method is presented in red color.

Our Haut dataset contains more complex and diverse CXR images than the two benchmark datasets. Our dataset segmentation model has achieved excellent results on two benchmark datasets through the above comparison. Figures 6 and 7 show the performance of our lung segmentation model in CXR images under different conditions, including clear lung field, fuzzy lung field, lung field blocked by foreign bodies, and lung field with segmentation failure.

### Metrics

Following are the five segmentation performance metrics we use: accuracy, sensitivity, specificity, dicecoefficient, and Jaccard Index. Semantic segmentation can be regarded as pixel-level classification. True Positive (TP): the model prediction is a positive example, which is a positive example. False Positive (FP): the model prediction is a positive example, but it is a negative example. False Negative (FN): the model prediction is a counterexample, but it is a positive example. True Negative (TN): the model prediction is a counterexample, it is a counterexample.

6 $$Accuracy = \frac{TP + TN}{{TP + TN + FP + FN}}$$

7 $$Specificity = \frac{TN}{{TN + FP}}$$

8 $$Sensitivity = \frac{TP}{{TP + FN}}$$

9 $$Dice = \frac{2TP}{{2TP + FP + FN }}$$

10 $$Jaccard\,\, Index = \frac{TP}{{TP + FP + FN}}$$

### Ethics statement

The study was approved by the Ethics Committee of the Henan University of Technology, all methods were carried out by relevant guidelines and regulations. Informed consent was obtained from all subjects and/or their legal guardian(s) and informed consent to publish was obtained from the doctors involved.

## Results

Ablations of the encoder and decoder sub-block approach on the JSRT, MC, and Haut are reported in Tables 1, 2, and 4. Tables 1, 2, and 4 list the accuracy, specificity, sensitivity, Dice coefficient, and Jaccard index for different methods on JSRT, MC, and Haut. From these results, it can be seen that our method 5(U-net architecture + Efficientnet-b4 encoder + two Residual blocks + LeakyReLU) has a higher mean value and lower standard deviation. Table 5 lists the mean Jaccard index of our method for lung segmentation in CXR images with different cases. Table 6 shows the research on lung segmentation by scholars in recent years and the results of this experiment. In general, the performance of our lung segmentation network is comparable to that of the excellent lung segmentation network proposed in the literature in recent years. That also encourages us to use the network to evaluate the lung segmentation performance of the Haut dataset.

**Table 4:** Lung segmentation results on the Haut dataset (mean ± standard deviation).

	Accuracy (%)	Specificity (%)	Sensitivity (%)	Dice coefficient (%)	Jaccard Index (%)
Method 1	96.72 ± 1.47	98.23 ± 0.80	93.00 ± 5.50	94.92 ± 1.63	87.42 ± 2.95
Method 2	97.23 ± 0.90	97.84 ± 0.56	94.82 ± 4.65	95.30 ± 1.38	88.25 ± 2.51
Method 3	97.45 ± 1.07	98.67 ± 0.30	96.44 ± 2.35	96.41 ± 0.94	91.33 ± 1.75
Method 4	99.24 ± 0.25	99.36 ± 0.29	99.22 ± 0.40	98.32 ± 0.27	96.17 ± 0.52
Method 5	**99.41 ± 0.20**	**99.52 ± 0.25**	**99.17 ± 0.37**	**98.84 ± 0.14**	**97.43 ± 0.27**
Method 6	99.40 ± 0.17	99.54 ± 0.24	99.14 ± 0.35	98.80 ± 0.13	97.40 ± 0.26

Significant values are in bold.

**Table 5:** CXR images with different cases have different segmentation scores.

	Mean Jaccard Index (%)
Healthy cases	97.86
Multiple diseases	96.52
Infiltration	97.14
Atelectasis	96.93
Effusion	96.42
Nodule	97.85
Mass	97.43
Pneumothorax	97.66
Cardiomegaly	97.70
Pleural thickening	97.85
Fibrosis	97.62
Consolidation	96.35
Emphysema	97.04
Edema	96.94
Pneumonia	97.52
Hernia	98.53

Multiple diseases mean a CXR image with two or more diseases.

**Table 6:** Comparison of results of the proposed method and recently related works.

Method	Dataset	Accuracy (%)	Specificity (%)	Sensitivity (%)	Dice coefficient (%)	Jaccard Index (%)
CNN + Morphological Optimization^20^	JSRT	98.5	–	–	99.2	98.5
Atrous Convolutions^14^	JSRT MC	–	–	–	98.0 on JSRT 96.4 on MC	96.1 on JSRT 94.1 on MC
Structured Edge Detector^30^	JSRT MC	–	–	–	97.6 on JSRT 95.6 on MC	95.8 on JSRT 93.5 on MC
Encoder-Decoder Structure^24^	JSRT	–	99.2	95.2	96.0	–
Improved FCN^17^	JSRT MC	98.9 on JSRT 97.4 on MC	–	–	–	95.8 on JSRT 91.7 on MC
Improved SegNet^18^	JSRT	98.7	–	–	–	95.1
U-Net^21^	JSRT MC	97.1 on JSRT 97.7 on MC	98.0 on JSRT 98.5 on MC	95.1 on JSRT 95.4 on MC	95.1 on JSRT 95.1 on MC	–
AlexNetand ResNet^23^	MC	96.9	96.7	97.5	94.0	88.0
Y. M. et al.^31^	JSRT MC	–	98.8 onJSRT 99.2 on MC	97.9 on JSRT 98.1 onMC	97.6 on JSRT 97.9 on MC	95.3 on JSRT 95.9 on MC
Our method	**JSRT**	**98.5**	**98.5**	**98.4**	**97.9**	**95.8**
	**MC**	**98.9**	**99.3**	**97.5**	**97.7**	**95.5**

Significant values are in bold.

### Comparison with other scholars

For the JSRT dataset, our model with a pre-trained Efficientnet-b4 base network achieved the accuracy of 98.5%, 98.5% of specificity, 98.4% of sensitivity, 97.9% of Dice coefficient, and 95.8% of the Jaccard Index using improved U-Net. Our model got an accuracy of 98.9%, 99.3% of specificity, 97.5% sensitivity, 97.7% dice coefficient, and 95.5% Jaccard index for the MC dataset. U-Net with a pre-trained Efficientnet-b4 base network provides advanced performance on the public datasets. Our model with a pre-trained Efficientnet-b4 network obtained an accuracy of 99.4% on the Haut dataset with 99.5% of specificity, 99.1% of sensitivity, 98.8% of dice coefficient, and 97.7% of Jaccard index with pre-trained Efficientnet-b4 base network, which is very encouraging and establishes the efficiency of our method. It also proves the effectiveness of our lung segmentation framework.

## Discussion

We summarized the previous studies of scholars and found that their work needs to be supplemented by later scholars. Most scholars are based on the JSRT and MC datasets, which do not contain lung segmentation in complex cases (severe pneumonia, foreign body shielding, lung deformation, etc.) Of course, some scholars try to label the NIH Chest X-ray dataset for lung segmentation22. But they do not verify the segmentation performance of the model on the benchmark dataset and do not summarize the segmentation scores of different CXR images. Our work complements these defects. To connect with the mainstream research on lung segmentation, we also did a series of experiments on JSRT and MC. In this study, we evaluated the efficacy of our model for lung segmentation on the JSRT, MC, and Haut datasets. Five segmentation performance indexes: Accuracy, Sensitivity, Specificity, Dice coefficient, and Jaccard index, are used to evaluate the model. We achieved excellent lung segmentation results. The segmentation score shows the reliability of our segmentation model. It is found that the transparency of the lung region, whether there is occlusion, and the shape of the lung will affect the results of lung segmentation to varying degrees. As shown in Fig. 7, it is difficult for the model to distinguish the lung region and lung boundary under the turbidity of the lung region caused by serious lung diseases. In addition, abnormal lung morphology is also difficult to segment. This is consistent with the results of other scholars.

The automatic lung segmentation model performs poorly in processing images of some diseases, such as pulmonary consolidation, lung effect, lung edema, and atelectasis. These diseases will make many exudates (tissue fluid, fibrin, etc.) fill the alveolar cavity and pleural cavity, resulting in lung densification and turbidity. It seriously affects the texture of the lung region in CXR images, so the automatic lung segmentation model may misinterpret these textures.

In addition, the automatic lung segmentation model is poor in dealing with severe lung deformation caused by congenital or acquired factors. Singh et al.^32^ recently published their lung segmentation study. Their scores far exceed those of previous scholars. But their data is absurd. Generally speaking, the Jaccard index is smaller than the Dice coefficient. But their result is just the opposite, which is very suspicious. So we didn't compare their experimental data.

## Conclusion

This paper proposes an accurate and robust automatic lung segmentation method based on U-Net architecture. This method uses the pre-trained Efficientnet-b4 as the encoder and uses the residual block and LeakyReLU to optimize the decoder. Our method achieves 95.8% and 95.5% Jaccard Index on JSRT and MC datasets, respectively. The accuracy is comparable to that obtained in the advanced literature in recent years. Based on the NIH Chest X-ray dataset, we randomly chose 2785 CXR images from it and invited experienced radiologists to mark their lung fields manually. These 2785 CXR images can be divided into 16 kinds of different situations. We use the above model to evaluate the segmentation performance in the Haut dataset. Achieved 97.4% of the overall Jaccard Index. However, the lung segmentation scores of different diseases are different. We found that chest radiograph segmentation scores were higher in healthy or mild conditions. The accuracy of lung segmentation is relatively low when the lung field is blurred, blocked by medical equipment, and severely deformed due to serious diseases. We also evaluated lung segmentation of specific illnesses.
